# Early hematoma expansion provides incremental prognostic information beyond admission characteristics for 6-month functional outcome after adult traumatic brain injury

**DOI:** 10.3389/fneur.2026.1942791

**Published:** 2026-09-29

**Authors:** Yinghao Yang, Changtao Liu, Rongjie Wu, Chengzhi Sun, Kang Xie, Jingshan Liang

**Affiliations:** 1 Department of Neurosurgery, Institute of Neuroscience, The Affiliated Lianyungang Hospital of Xuzhou Medical University, The First People's Hospital of Lianyungang, Lianyungang, China; 2 Department of Neurosurgery, The First People's Hospital of Wuhu, Anhui, China

**Keywords:** computed tomography, functional outcome, hematoma expansion, prognosis, traumatic brain injury

## Abstract

**Background:**

Early hematoma expansion (HE) reflects interval progression of traumatic intracranial hemorrhage, but its association with long-term outcome and its prognostic contribution beyond admission characteristics remain incompletely defined. We evaluated whether HE was independently associated with 6-month functional outcome and provided incremental prognostic information beyond admission characteristics.

**Methods:**

This single-center retrospective study included 640 adults with traumatic brain injury, including 285 with HE. HE was determined from the first two in-hospital head computed tomography scans obtained within 48 h after injury and was defined as a new intracranial hemorrhagic lesion or an increase of >3 ml in total hemorrhage volume. Six-month outcome was assessed using the Glasgow Outcome Scale (1–3 unfavorable; 4–5 favorable). HE was added to a study-specific baseline model comprising age, admission Glasgow Coma Scale score, D-dimer, baseline hematoma volume, cerebral contusion, diffuse axonal injury, frontal lobe involvement, and long-bone fracture.

**Results:**

At 6 months, 145 patients (22.7%) had an unfavorable outcome. HE was independently associated with a lower likelihood of favorable outcome (OR, 0.20; 95% CI, 0.10–0.42). Adding HE reduced the Akaike information criterion from 392.767 to 365.908 (likelihood-ratio *P* < 0.001) and increased the area under the curve from 0.920 to 0.929 (ΔAUC = 0.009; DeLong *P* = 0.0289). Prognostic heterogeneity remained among HE-positive patients.

**Conclusions:**

Early HE is independently associated with 6-month functional outcome in adult patients with TBI. As a readily obtainable finding on routine serial CT, HE may help refine early prognostic assessment in patients with TBI.

## 1 Introduction

Traumatic brain injury (TBI) is a leading cause of death and long-term disability worldwide, imposing a substantial and persistent burden on patients, families, and healthcare systems (1, 2). Early identification of patients at high risk of poor outcomes is essential for optimizing clinical decision-making and guiding individualized management strategies (3). Consequently, the development of accurate, interpretable, and clinically applicable prognostic models remains a major objective in TBI research. Current prognostic assessment primarily relies on baseline clinical and radiological variables obtained at a single time point. However, these static measurements cannot adequately capture the dynamic progression of secondary brain injury that continues to evolve during the early post-traumatic period (4, 5). Although prediction models incorporating longitudinal data and advanced computational algorithms have shown encouraging results, their widespread clinical implementation remains limited by complex analytical workflows, intensive data requirements, and limited interpretability (6–8).

Early hematoma expansion (HE), a dynamic radiological manifestation of ongoing intracranial hemorrhagic progression, can be readily identified using routine serial computed tomography (CT). HE reflects continued bleeding, increasing mass effect, and evolving secondary brain injury, all of which are closely related to neurological deterioration (9–16). Previous studies have primarily focused on identifying predictors of HE or developing models to estimate its occurrence (8, 13, 15, 17, 18). In contrast, whether HE remains associated with functional outcome after accounting for admission clinical, laboratory, and radiographic characteristics remains unclear. Furthermore, although patients with HE are generally considered to have a worse prognosis, substantial heterogeneity in functional outcomes persists within this population, and factors contributing to this heterogeneity remain poorly understood.

In the present study, HE was defined using the first two in-hospital head CT examinations obtained within 48 h after injury and was evaluated as an early dynamic imaging biomarker in adult patients with TBI. We aimed to investigate the independent association between early HE and 6-month functional outcome, determine whether HE provides incremental prognostic information beyond admission characteristics, and further explore prognostic heterogeneity among patients who had developed HE.

## 2 Materials and methods

### 2.1 Study population

A total of 952 consecutive adult patients with traumatic brain injury (TBI) admitted to the First People's Hospital of Lianyungang between March 1, 2021, and March 31, 2023, were screened for eligibility. Patients were excluded if they underwent brain surgery before the second CT examination (*n* = 59), if the interval between injury onset and the second CT scan exceeded 48 h (*n* = 41), if the baseline CT scan had been performed at another institution (*n* = 124), if they were younger than 18 years (*n* = 34), or if follow-up data were unavailable (*n* = 54). Ultimately, 640 patients were included in the final analysis, including 285 patients with hematoma expansion (HE). This requirement ensured that HE was evaluated using serial in-hospital CT examinations before surgical alteration of hematoma volume. The patient selection process is illustrated in Figure 1. The study was approved by the Medical Ethics Committee of The First People's Hospital of Lianyungang (approval No. LW-20240627001-01).

**Figure 1:**
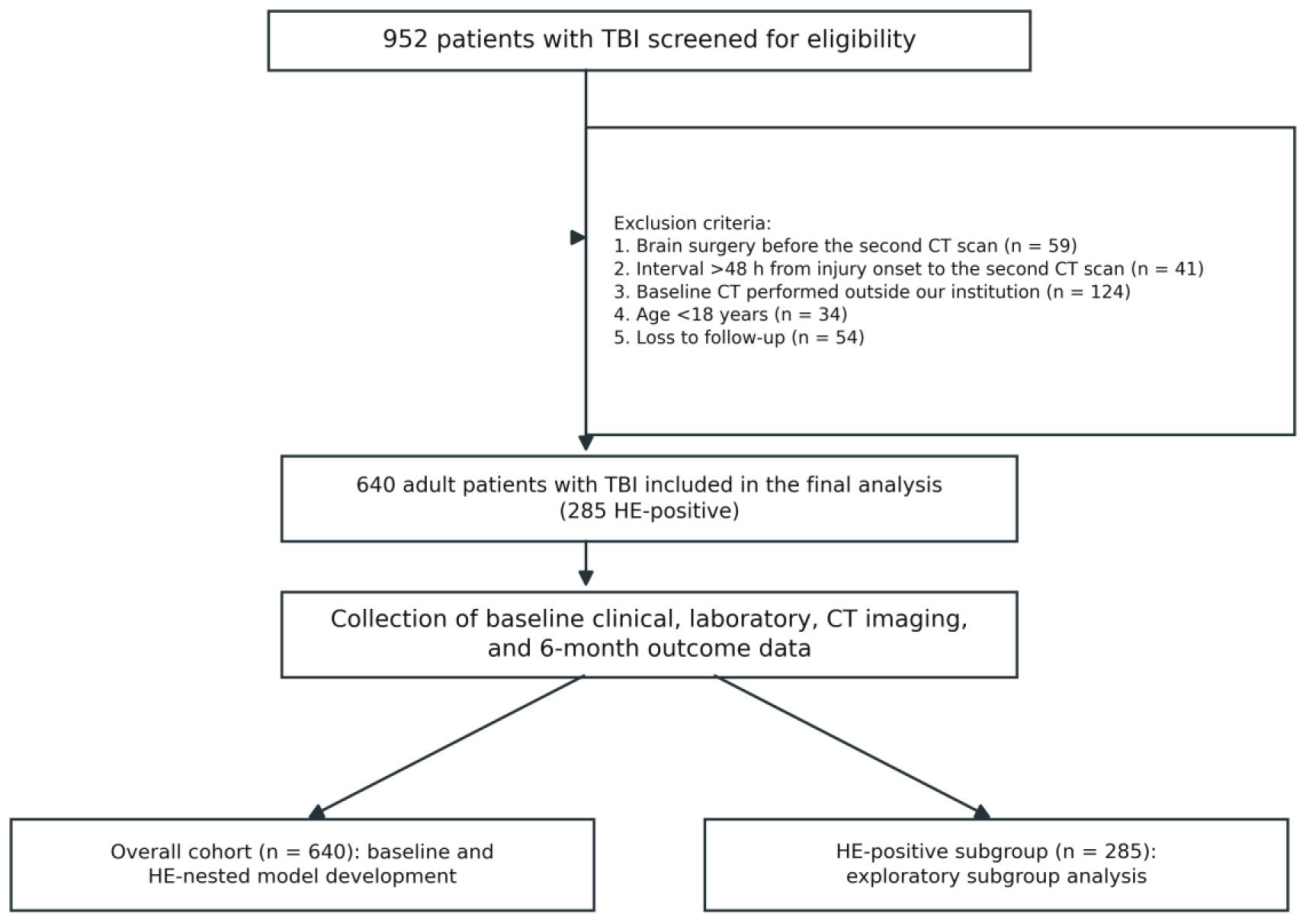
Study flowchart of patient selection and analytical framework. A total of 952 patients with traumatic brain injury (TBI) were screened for eligibility. After exclusion of patients who underwent brain surgery before the follow-up CT scan, had an interval of >48 h from injury onset to the follow-up CT scan, had the baseline CT scan performed outside our institution, were younger than 18 years, or were lost to follow-up, 640 adult patients with TBI were included in the final analysis, of whom 285 were hematoma expansion (HE)-positive. Baseline clinical, laboratory, CT imaging, and 6-month outcome data were collected. The overall cohort was used for development of the baseline model and the nested HE model, and the HE-positive subgroup was used for exploratory subgroup analysis.

### 2.2 Clinical characteristics and outcome assessment

Clinical variables were considered as candidate baseline characteristics because they were routinely obtained during initial TBI assessment. Baseline clinical data collected at admission included demographic characteristics (age and sex), medical history (including hypertension and diabetes mellitus), vital signs (heart rate, body temperature, respiratory rate, systolic and diastolic blood pressure, bilateral pupil diameter), and the Glasgow Coma Scale (GCS) score (19). Initial laboratory parameters included complete blood count indices [white blood cell count (WBC), neutrophil proportion (NE), platelet count (PLT), and hemoglobin (Hb)], serum biochemical parameters (glucose, sodium, potassium, and calcium), and coagulation markers [prothrombin time (PT), activated partial thromboplastin time (APTT), fibrinogen, D-dimer, and fibrin degradation products (FDP)].

Functional outcome was evaluated 6 months after injury using the Glasgow Outcome Scale (GOS) (20). A GOS score of 1–3 was defined as an unfavorable outcome, whereas a score of 4–5 was considered a favorable outcome.

### 2.3 CT acquisition

All patients underwent non-contrast head CT according to the institutional emergency imaging protocol using a 32-slice CT scanner (Definition AS, Siemens Healthcare Solutions, Forchheim, Germany). Scans were obtained from the skull base to the vertex using the following acquisition parameters: tube voltage 120 kVp, tube current 380 mAs, and slice thickness/interval of 5 mm.

### 2.4 Hematoma volume measurement and definition of hematoma expansion

Intracranial hematoma volume was measured on the first two in-hospital CT scans using 3D Slicer (version 5.0.3). HE was defined as either the appearance of a new intracranial hemorrhagic lesion on follow-up CT or an increase of more than 3 ml in total intracranial hemorrhage volume compared with the baseline CT scan (9, 11). Eligibility required completion of the follow-up CT examination within 48 h after injury (10, 11). This window was selected to capture the principal early period of post-traumatic hemorrhagic evolution while accommodating variation in progression across lesion types. The absolute >3 ml threshold was selected based on previous traumatic hematoma expansion studies and to avoid instability from relative changes in small baseline hematomas.

Hematoma segmentation was independently performed by two attending neurosurgeons. The final hematoma volume was calculated as the mean of the two measurements. If the discrepancy between measurements exceeded 2 ml, the final volume was adjudicated by a senior neurosurgeon. Representative segmentation images are shown in Supplementary Figure 1.

### 2.5 Statistical analysis

Multivariable logistic regression was used to develop prognostic models for 6-month functional outcome. Candidate baseline variables comprised routinely available admission clinical, laboratory and imaging characteristics. Variables associated with 6-month functional outcome in univariable analyses at *P* < 0.05 were entered into multivariable logistic regression. Variables remaining statistically significant at *P* < 0.05 in the multivariable model were retained in the final study-specific baseline model. HE was subsequently incorporated into the baseline model to construct a nested model, allowing evaluation of the incremental prognostic value of HE beyond the admission characteristics included in the baseline model (21). A best-subset sensitivity analysis was performed in the overall cohort of 640 patients using AIC, BIC, and AUC.

To explore whether patients with HE remained prognostically heterogeneous, we performed an exploratory analysis restricted to the HE-positive cohort to identify admission characteristics that remained associated with 6-month functional outcome after HE had occurred. This analysis was exploratory and intended to characterize prognostic heterogeneity rather than to develop an externally applicable prediction model.

All statistical analyses were performed using R software (version 4.3.1). Continuous variables were assessed for normality and are presented as medians with interquartile ranges (IQR) because the included continuous variables were non-normally distributed. Categorical variables are expressed as number (percentage). Group comparisons were performed using the Mann–Whitney *U* test for continuous variables and the Pearson χ^2^ test or Fisher's exact test for categorical variables, as appropriate. All variables included in the final baseline model, nested HE model, and HE-positive subgroup model were complete among the 640 patients. No imputation was performed for variables included in the primary regression models because these variables were complete among the 640 patients. All statistical tests were two-sided, and a *P* value < 0.05 was considered statistically significant. Comparisons according to HE status were descriptive and were not used for variable selection or model construction.

Multivariable logistic regression was used to estimate odds ratios (ORs) and corresponding 95% confidence intervals (CIs). Model discrimination was evaluated using receiver operating characteristic (ROC) curves and the area under the ROC curve (AUC). Differences in AUC between models were compared using the DeLong test. Improvements in overall model performance were assessed by changes in the Akaike information criterion (AIC) together with the likelihood ratio test. Model calibration was evaluated using calibration plots, and clinical utility was assessed by decision curve analysis (DCA). Internal validation was performed using 1,000 bootstrap resamples to obtain optimism-corrected AUCs and calibration estimates (22). As a sensitivity analysis, the interval between the first and second in-hospital CT examinations was forced into both models. Values >48 h were truncated at 48 h, and four erroneous non-positive interval values were replaced using the corresponding HE-group mean, after which model fit and discrimination were reassessed.

The same modeling framework was applied in the HE-positive subgroup. All models were specified to predict favorable functional outcome; therefore, an OR > 1 indicated a higher likelihood of favorable outcome, whereas an OR < 1 indicated a lower likelihood of favorable outcome.

## 3 Results

### 3.1 Baseline characteristics of the study population

A total of 640 adult patients with traumatic brain injury (TBI) were included in the final analysis. Baseline clinical, laboratory, and radiographic characteristics according to HE status are descriptively summarized in Supplementary Table 1. At 6 months, 145 patients (22.7%) had an unfavorable functional outcome, whereas 495 (77.3%) achieved a favorable outcome (Table 1).

**Table 1:** Baseline characteristics of adult patients with traumatic brain injury according to 6-month functional outcome.

Variable	Total (*n* = 640)	Unfavorable (*n* = 145)	Favorable (*n* = 495)	Statistic	*P* value
Age, years	58.00 (47.00, 67.00)	63.00 (56.00, 70.00)	56.00 (43.50, 66.00)	*Z* = −5.24	< 0.001
Left pupil diameter, mm	3.00 (3.00, 3.00)	3.00 (2.50, 3.00)	3.00 (3.00, 3.00)	*Z* = −3.14	0.002
Right pupil diameter, mm	3.00 (3.00, 3.00)	3.00 (2.50, 3.00)	3.00 (3.00, 3.00)	*Z* = −0.87	0.382
GCS score	14.00 (11.00, 15.00)	8.00 (6.00, 13.00)	15.00 (14.00, 15.00)	*Z* = −13.30	< 0.001
Systolic blood pressure, mmHg	140.00 (127.00, 153.00)	146.00 (134.00, 163.00)	139.00 (126.00, 152.00)	*Z* = −3.74	< 0.001
Diastolic blood pressure, mmHg	82.00 (74.00, 90.00)	82.00 (75.00, 93.00)	81.00 (73.00, 90.00)	*Z* = −0.85	0.395
Heart rate, beats/min	78.00 (70.00, 86.25)	78.00 (67.00, 91.00)	78.00 (70.00, 86.00)	*Z* = −0.05	0.963
Body temperature, °C	36.60 (36.40, 36.90)	36.60 (36.50, 37.00)	36.60 (36.40, 36.85)	*Z* = −1.67	0.094
Glucose, mmol/L	6.68 (5.54, 8.16)	7.76 (6.61, 10.04)	6.29 (5.40, 7.65)	*Z* = −7.26	< 0.001
Potassium, mmol/L	3.81 (3.56, 4.03)	3.71 (3.45, 3.99)	3.84 (3.58, 4.04)	*Z* = −2.61	0.009
Sodium, mmol/L	138.85 (137.10, 141.00)	139.00 (137.20, 141.90)	138.70 (137.10, 140.70)	*Z* = −2.05	0.041
Calcium, mmol/L	2.19 (2.10, 2.26)	2.14 (2.05, 2.23)	2.19 (2.12, 2.27)	*Z* = −4.10	< 0.001
PT, s	11.80 (11.20, 12.50)	12.10 (11.40, 13.10)	11.70 (11.20, 12.40)	*Z* = −4.27	< 0.001
APTT, s	28.30 (26.45, 30.40)	28.50 (26.40, 29.90)	28.25 (26.50, 30.50)	*Z* = −0.29	0.773
Fibrinogen, g/L	2.62 (2.21, 3.08)	2.44 (2.00, 3.27)	2.65 (2.26, 3.04)	*Z* = −1.54	0.124
D-dimer	3,015.00 (869.00, 7,219.50)	7,485.00 (2,753.00, 18,914.00)	2,442.00 (679.00, 5,607.50)	*Z* = −8.67	< 0.001
FDP	23.45 (7.43, 64.67)	77.25 (21.19, 169.99)	17.72 (5.99, 47.61)	*Z* = −8.93	< 0.001
WBC	12.32 (9.48, 15.81)	14.09 (10.81, 17.73)	12.03 (9.27, 15.22)	*Z* = −4.10	< 0.001
Neutrophils, %	87.50 (81.80, 90.90)	88.95 (84.10, 91.45)	87.20 (80.70, 90.70)	*Z* = −3.24	0.001
Hemoglobin, g/L	132.00 (119.00, 144.00)	125.00 (110.00, 138.00)	134.00 (122.00, 146.00)	*Z* = −5.97	< 0.001
Platelet count	195.00 (155.75, 236.00)	188.00 (146.00, 231.00)	197.00 (157.50, 236.00)	*Z* = −1.52	0.13
Time to baseline CT, h	2.00 (1.50, 2.00)	1.50 (1.50, 2.00)	2.00 (1.50, 2.00)	*Z* = −6.37	< 0.001
Baseline hematoma volume, ml	3.18 (1.70, 6.16)	6.14 (3.46, 10.86)	2.65 (1.54, 4.74)	*Z* = −8.77	< 0.001
Epidural hematoma, *n* (%)	161 (25.16)	38 (26.21)	123 (24.85)	χ^2^ = 0.11	0.74
Diffuse axonal injury, *n* (%)	51 (7.97)	24 (16.55)	27 (5.45)	χ^2^ = 18.83	< 0.001
Subdural hematoma, *n* (%)	370 (57.81)	110 (75.86)	260 (52.53)	χ^2^ = 25.04	< 0.001
Subarachnoid hemorrhage, *n* (%)	450 (70.31)	127 (87.59)	323 (65.25)	χ^2^ = 26.80	< 0.001
Long–bone fracture, *n* (%)	170 (26.56)	60 (41.38)	110 (22.22)	χ^2^ = 21.10	< 0.001
Skull fracture, *n* (%)	412 (64.38)	104 (71.72)	308 (62.22)	χ^2^ = 4.42	0.036
**Frontal lobe involvement**, ***n*** **(%)**					
Diffuse/bilateral	156 (24.38)	63 (43.45)	93 (18.79)	χ^2^ = 53.19	< 0.001
Left	121 (18.91)	27 (18.62)	94 (18.99)		
None	208 (32.50)	17 (11.72)	191 (38.59)		
Right	155 (24.22)	38 (26.21)	117 (23.64)		
**Parietal lobe involvement**, ***n*** **(%)**					
Diffuse/bilateral	50 (7.81)	20 (13.79)	30 (6.06)	χ^2^ = 25.04	< 0.001
Left	116 (18.12)	34 (23.45)	82 (16.57)		
None	366 (57.19)	58 (40.00)	308 (62.22)		
Right	108 (16.88)	33 (22.76)	75 (15.15)		
**Occipital lobe involvement**, ***n*** **(%)**					
Diffuse/bilateral	10 (1.56)	4 (2.76)	6 (1.21)	χ^2^ = 7.60	0.055
Left	77 (12.03)	18 (12.41)	59 (11.92)		
None	492 (76.88)	102 (70.34)	390 (78.79)		
Right	61 (9.53)	21 (14.48)	40 (8.08)		
**Temporal lobe involvement**, ***n*** **(%)**					
Diffuse/bilateral	128 (20.00)	47 (32.41)	81 (16.36)	χ^2^ = 23.48	< 0.001
Left	150 (23.44)	31 (21.38)	119 (24.04)		
None	173 (27.03)	23 (15.86)	150 (30.30)		
Right	189 (29.53)	44 (30.34)	145 (29.29)		
**Sex**, ***n*** **(%)**					
Female	223 (34.84)	49 (33.79)	174 (35.15)	χ^2^ = 0.09	0.763
Male	417 (65.16)	96 (66.21)	321 (64.85)		
**Deep structure involvement**, ***n*** **(%)**					
No	510 (79.69)	97 (66.90)	413 (83.43)	χ^2^ = 18.95	< 0.001
Yes	130 (20.31)	48 (33.10)	82 (16.57)		

Continuous variables are presented as median (IQR), and categorical variables as *n* (%). Unfavorable outcome was defined as a 6-month Glasgow Outcome Scale (GOS) score of 1–3, and favorable outcome as a GOS score of 4–5. *P* values were calculated using the Mann–Whitney *U* test for continuous variables and Pearson's chi-square test or Fisher's exact test for categorical variables, as appropriate. For lobar involvement pattern, categories were diffuse/bilateral involvement, left-sided involvement, no involvement, and right-sided involvement.

APTT, activated partial thromboplastin time; GCS, Glasgow coma scale; CT, computed tomography; DAI, diffuse axonal injury; FDP, fibrin degradation products; GOS, Glasgow outcome scale; HE, hematoma expansion; PT, prothrombin time.

Compared with patients who achieved a favorable outcome, those with an unfavorable outcome were significantly older [63.0 (56.0–70.0) vs. 56.0 (43.5–66.0) years, *P* < 0.001] and had lower admission Glasgow Coma Scale (GCS) scores [8.0 (6.0–13.0) vs. 15.0 (14.0–15.0), *P* < 0.001]. The unfavorable outcome group also had significantly higher frequencies of cerebral contusion, diffuse axonal injury, subdural hematoma, subarachnoid hemorrhage, deep structure involvement, skull fracture, long bone fracture, and hematoma expansion (HE) (all *P* < 0.05). In contrast, the incidence of epidural hematoma did not differ significantly between groups (*P* = 0.740).

Regarding lesion distribution, patients with unfavorable outcomes exhibited more extensive frontal, parietal, and temporal lobe involvement, a higher frequency of bilateral/diffuse lobar involvement, involvement of a greater number of cerebral lobes, and more frequent deep structure involvement (all *P* < 0.001). Occipital lobe involvement was comparable between the two groups (*P* = 0.055).

### 3.2 Prognostic performance of the baseline and nested HE models

The multivariable logistic regression results for the baseline model and the nested HE model are presented in Supplementary Table 2.

After incorporation of HE into the baseline model, HE remained independently associated with a lower likelihood of favorable 6-month functional outcome. Older age, higher D-dimer level, larger baseline hematoma volume, and long bone fracture also remained independently associated with unfavorable outcome, whereas a higher admission GCS score was independently associated with favorable outcome. Compared with diffuse frontal lobe involvement, the absence of frontal lobe involvement was associated with a higher probability of favorable outcome. Notably, cerebral contusion and diffuse axonal injury were no longer independently associated with outcome after HE was incorporated into the model. Receiver operating characteristic (ROC) curves for both models are shown in Figure 2.

**Figure 2:**
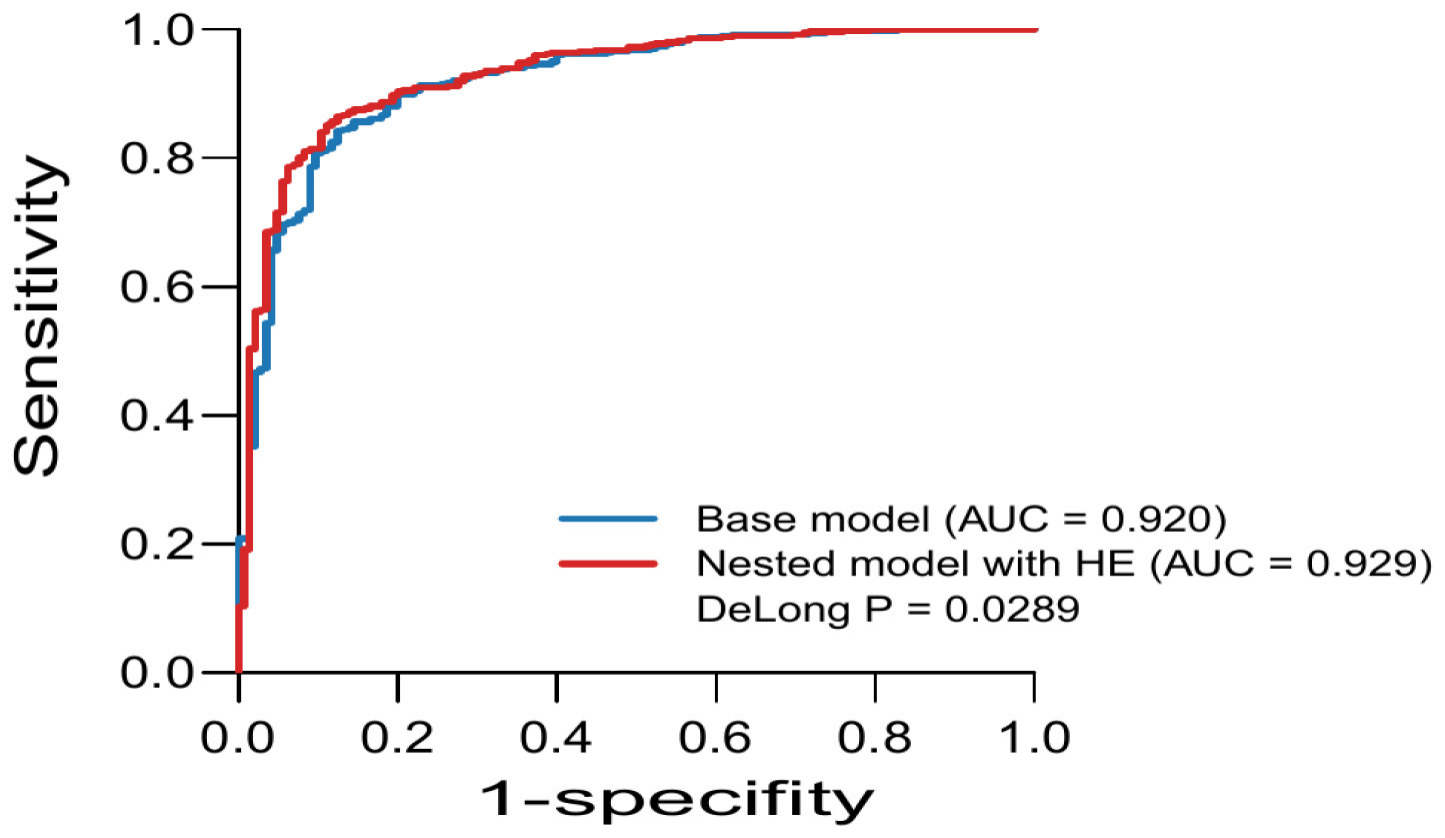
Receiver operating characteristic curves of the baseline model and the nested HE model for predicting favorable 6-month functional outcome in the overall cohort.

Incorporation of HE improved model fit and produced a modest increase in discrimination. The Akaike information criterion (AIC) decreased from 392.767 to 365.908 (ΔAIC = 26.859), and the likelihood ratio test confirmed a significant improvement in model fit (χ^2^ = 28.859, *P* < 0.001) (Table 2). Likewise, discrimination improved, with the area under the ROC curve (AUC) increasing from 0.920 to 0.929 (ΔAUC = 0.009; DeLong *P* = 0.0289) (Supplementary Table 3), with corresponding optimism-corrected AUCs of 0.913 and 0.922 (Table 2).

**Table 2:** Predictive performance of the baseline model and the nested HE model for 6-month favorable functional outcome in the overall cohort.

Metric	Baseline model	Nested HE model
AUC (95% CI)	0.92 (0.89–0.95)	0.93 (0.91–0.95)
Optimism-corrected AUC (95% CI)	0.91 (0.88–0.94)	0.92 (0.90–0.94)
Accuracy (95% CI)	0.85 (0.82–0.88)	0.87 (0.84–0.89)
Sensitivity (95% CI)	0.84 (0.81–0.87)	0.86 (0.83–0.89)
Specificity (95% CI)	0.88 (0.82–0.93)	0.88 (0.82–0.93)
PPV (95% CI)	0.96 (0.94–0.98)	0.96 (0.94–0.98)
NPV (95% CI)	0.62 (0.55–0.69)	0.65 (0.59–0.72)
Optimal cutoff	0.795	0.764

AUC, area under the receiver operating characteristic curve; CI, confidence interval; HE, hematoma expansion.

Using the optimal cutoff identified by the Youden index, the nested HE model achieved an AUC of 0.929 (95% CI, 0.906–0.953), with an accuracy of 0.867, sensitivity of 0.865, specificity of 0.876, positive predictive value of 0.960, and negative predictive value of 0.655. Calibration performance is illustrated in Figure 3, whereas decision curve analysis and bootstrap-corrected calibration are presented Supplementary Figures 2, 3, respectively.

**Figure 3:**
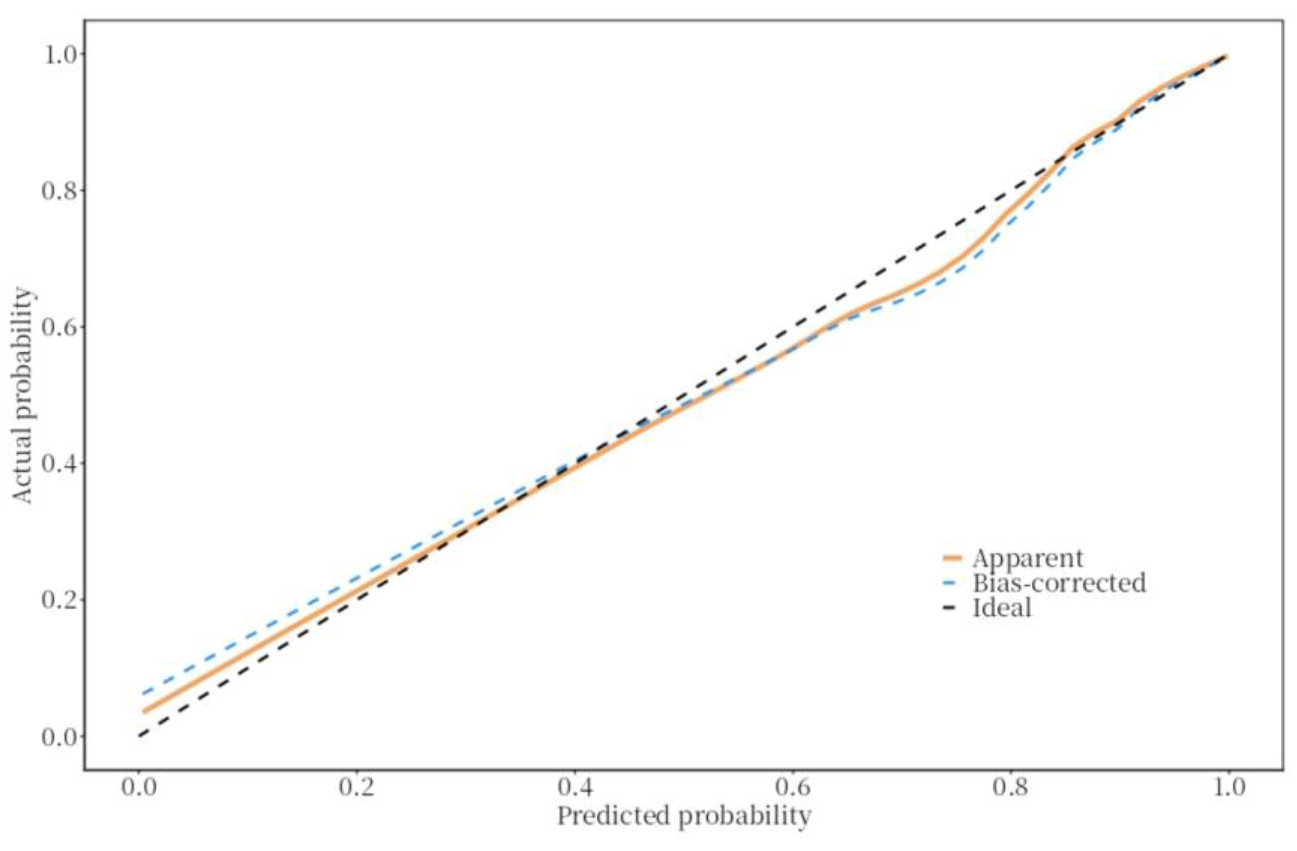
Calibration curve of the nested HE model for predicting favorable 6-month functional outcome in the overall cohort. Calibration plot of the nested HE model for predicting favorable 6-month functional outcome in the overall cohort. Predicted probability is shown on the x-axis and observed probability on the y-axis. The figure presents the calibration performance of the nested HE model in the derivation cohort.

As an additional best-subset sensitivity analysis in the overall cohort of 640 patients, AIC-based best-subset selection selected the same baseline model as the primary analysis, whereas BIC-based selection yielded a more parsimonious model with lower discrimination. After HE was added, model performance improved consistently, supporting the robustness of the selected baseline model and the incremental prognostic contribution of HE (Supplementary Table 4).

In a timing-adjusted sensitivity analysis, additional adjustment for the interval between the baseline and follow-up CT examinations did not materially alter the association between HE and 6-month functional outcome or its incremental prognostic performance. The results were consistent with the primary analysis (Supplementary Table 5).

### 3.3 Exploratory subgroup analysis in HE-positive patients

An exploratory analysis was subsequently performed in the 285 patients with HE. Multivariable logistic regression results are presented in Table 3.

**Table 3:** Multivariable logistic regression analysis of factors associated with favorable 6-month functional outcome in HE-positive patients.

Variable	β	SE	Z	*P* value	OR (95% CI)
Intercept	1.75	0.98	1.78	0.075	5.73 (0.84–39.27)
Diffuse axonal injury					
N					1.00 (Reference)
Y	−1.22	0.55	−2.23	0.026	0.30 (0.10–0.86)
Long bone fracture present					
N					1.00 (Reference)
Y	−1.34	0.37	−3.64	< 0.001	0.26 (0.13–0.54)
Frontal involvement					
D					1.00 (Reference)
L	0.53	0.43	1.23	0.219	1.69 (0.73–3.93)
N	1.41	0.56	2.54	0.011	4.10 (1.38–12.18)
R	0.52	0.47	1.11	0.268	1.68 (0.67–4.20)
Age years	−0.05	0.01	−3.82	< 0.001	0.95 (0.92–0.98)
GCS	0.29	0.05	5.56	< 0.001	1.33 (1.20–1.47)
D-dimer	−0.01	0	−2.9	0.004	0.99 (0.99–0.99)
Baseline hematoma volume, ml	−0.18	0.05	−3.87	< 0.001	0.83 (0.76–0.91)

HE, hematoma expansion; GCS, Glasgow coma scale; OR, odds ratio; CI, confidence interval.

Within the HE-positive cohort, diffuse axonal injury, long-bone fracture, older age, higher D-dimer level, and larger baseline hematoma volume were independently associated with unfavorable functional outcome, whereas a higher admission GCS score remained independently associated with favorable outcome. Compared with diffuse frontal lobe involvement, the absence of frontal lobe involvement was associated with a significantly higher likelihood of favorable outcome, whereas neither left-sided nor right-sided frontal lobe involvement showed a significant association. Discrimination metrics for this exploratory HE-positive subgroup analysis are summarized in Supplementary Table 6.

## 4 Discussion

In this study, we demonstrated that early HE, identified on routine serial CT within 48 h after injury, was independently associated with 6-month functional outcome in adult patients with TBI. More importantly, incorporation of HE into the study-specific baseline model improved model fit and yielded a modest but statistically supported increase in discrimination, indicating that HE provided additional prognostic information beyond the admission characteristics included in the present analysis (23). Furthermore, clinically meaningful heterogeneity remained evident among patients with HE, suggesting that HE-positive patients should not be regarded as a homogeneous high-risk population (23, 24). The best-subset sensitivity analysis further supported the primary model specification, indicating that the main findings were not driven by a single variable-selection approach.

The association between HE and functional outcome is biologically plausible. TBI is characterized by evolving secondary brain injury, during which intracranial hemorrhagic lesions may continue to progress after the initial insult (14, 24). Consequently, baseline clinical and imaging characteristics alone may not fully capture the subsequent evolution of injury. HE reflects ongoing post-traumatic hemorrhagic progression and evolving secondary brain injury, thereby providing dynamic information on disease progression beyond the initial injury severity (11, 25). Accordingly, HE was evaluated in the present study not merely as an accompanying radiological finding but as a dynamic imaging biomarker incorporated into a hierarchical prognostic framework.

An important finding of this study is that the prognostic value of HE extends beyond its simple presence or absence. Because HE can be identified from routine serial CT examinations obtained during standard clinical care, it represents a clinically accessible source of dynamic prognostic information without requiring additional specialized imaging acquisition or intensive longitudinal data collection (26). Comparison between the overall cohort and the HE-positive subgroup demonstrated consistent associations for several established prognostic factors, including age, admission Glasgow Coma Scale score, D-dimer level, baseline hematoma volume, and long bone fracture. Although long-bone fracture is not a direct marker of intracranial injury, concomitant major skeletal injury may reflect the broader burden of trauma and has previously been associated with worse outcomes after TBI (27). In contrast, cerebral contusion and diffuse axonal injury lost statistical significance after HE was incorporated into the overall model. This attenuation suggests partial overlap in the prognostic information captured by HE and these baseline imaging variables, with HE showing a stronger association with outcome in the combined model (23, 26). Rather than replacing conventional imaging markers, HE appears to provide complementary dynamic prognostic information during the early phase after TBI.

Established prognostic models such as IMPACT and CRASH provide well-validated frameworks for long-term outcome prediction after TBI (23). Our findings extend this framework by showing that HE identified on routine serial CT provides additional prognostic information beyond admission characteristics and may serve as a dynamic adjunct to established assessment. Although the increase in AUC was modest, decision curve analysis (Supplementary Figure 2) suggested potential net benefit of the nested HE model over default strategies across a range of threshold probabilities. Because HE detection may be influenced by the interval between baseline and follow-up CT, we performed a timing-adjusted sensitivity analysis: the association between HE and 6-month outcome and its incremental discrimination remained stable after adjustment, suggesting that these findings were not primarily driven by differences in CT timing. Nevertheless, HE-positive patients remained prognostically heterogeneous. Age, admission GCS score, D-dimer, baseline hematoma volume, diffuse axonal injury, and long-bone fracture remained independently associated with outcome, indicating that HE should be interpreted within the broader clinical and injury context rather than as a stand-alone prognostic marker (27–32).

Several limitations should be acknowledged. First, this was a retrospective single-center study without external validation, and the requirement for two serial in-hospital CT examinations before hematoma evacuation may limit the generalizability of the findings. Second, the definition of HE and the 48-h observation window were pragmatic choices based on routine serial CT practice and previous traumatic HE studies; alternative thresholds, including combined absolute-and-relative definitions, may yield different HE classifications. Third, formal inter-rater reliability statistics could not be calculated because individual rater-level volumetric records were not retained. Fourth, although the association of HE with outcome remained stable after adjustment for the baseline-to-follow-up CT interval, the timing of repeat CT was not protocolized and residual indication- or timing-related bias cannot be excluded. Fifth, the baseline model did not include all established radiographic features, such as midline shift or basal cistern status, or complete established prognostic frameworks such as IMPACT and CRASH. Therefore, the incremental prognostic information provided by HE should be interpreted in relation to the admission characteristics evaluated in our cohort. Finally, the exploratory findings within the HE-positive subgroup warrant further validation in larger multicenter cohorts.

Collectively, these findings support the prognostic relevance of early interval changes on routine serial CT in TBI. HE may represent a clinically accessible dynamic marker that complements admission-based assessment and refines interpretation of subsequent outcome risk.

## 5 Conclusion

Early hematoma expansion is independently associated with 6-month functional outcome after adult TBI and provides incremental prognostic information beyond admission characteristics. As a readily obtainable finding on routine serial CT, HE may help refine early prognostic assessment after TBI.

## Data Availability

The original contributions presented in the study are included in the article/Supplementary material, further inquiries can be directed to the corresponding authors.
