# Supplementary material for: Early hematoma expansion provides incremental prognostic information beyond admission characteristics for 6-month functional outcome after adult traumatic brain injury
Source: Front Neurol. 2026 Sep 29;17:1942791. doi: 10.3389/fneur.2026.1942791 (PMC13657924; doi:10.3389/fneur.2026.1942791)
Supplement: Supplementary file 1 [file Supplementary_File_1.docx]

**Early hematoma expansion provides incremental prognostic information beyond admission characteristics for 6-month functional outcome after adult traumatic brain injury**

**Supplementary Table and Figure Legends**

**Supplementary Table 1 Characteristics of adult patients with traumatic brain injury according to hematoma expansion status**

| **Variable** | **Total (n = 640)** | **No HE (n = 355)** | **HE (n = 285)** | **P value** |
| --- | --- | --- | --- | --- |
| **Clinical characteristics** | | | | |
| Age, years | 58.00 (47.00, 67.00) | 57.00 (45.00, 67.00) | 58.00 (50.00, 68.00) | 0.061 |
| Left pupil diameter, mm (valid n=626) | 3.00 (3.00, 3.00) | 3.00 (3.00, 3.00) | 3.00 (3.00, 3.00) | 0.019 |
| Right pupil diameter, mm (valid n=619) | 3.00 (3.00, 3.00) | 3.00 (3.00, 3.00) | 3.00 (3.00, 3.00) | 0.099 |
| GCS score | 14.00 (11.00, 15.00) | 15.00 (14.00, 15.00) | 13.00 (8.00, 14.00) | <0.001 |
| Systolic blood pressure, mmHg | 140.00 (127.00, 153.00) | 136.00 (125.00, 151.00) | 144.00 (130.00, 155.00) | <0.001 |
| Diastolic blood pressure, mmHg | 82.00 (74.00, 90.00) | 81.00 (72.00, 91.00) | 83.00 (75.00, 90.00) | 0.385 |
| Heart rate, beats/min | 78.00 (70.00, 86.25) | 78.00 (70.00, 86.00) | 78.00 (70.00, 87.00) | 0.502 |
| Body temperature, °C | 36.60 (36.40, 36.90) | 36.60 (36.40, 36.80) | 36.60 (36.50, 36.90) | 0.207 |
| Sex, n (%) |  |  |  | 0.142 |
| Female | 223 (34.84) | 133 (37.46) | 90 (31.58) |  |
| Male | 417 (65.16) | 222 (62.54) | 195 (68.42) |  |
| **Laboratory characteristics** | | | | |
| Glucose, mmol/L (valid n=639) | 6.68 (5.54, 8.16) | 6.07 (5.32, 7.61) | 7.29 (6.17, 8.73) | <0.001 |
| Potassium, mmol/L | 3.81 (3.56, 4.03) | 3.86 (3.62, 4.08) | 3.76 (3.50, 3.99) | 0.001 |
| Sodium, mmol/L | 138.85 (137.10, 141.00) | 138.80 (137.10, 140.85) | 138.90 (137.10, 141.10) | 0.621 |
| Calcium, mmol/L | 2.19 (2.10, 2.26) | 2.20 (2.12, 2.28) | 2.16 (2.08, 2.24) | <0.001 |
| PT, s | 11.80 (11.20, 12.50) | 11.70 (11.20, 12.40) | 11.90 (11.30, 12.70) | 0.008 |
| APTT, s (valid n=639) | 28.30 (26.45, 30.40) | 28.20 (26.50, 30.80) | 28.40 (26.20, 30.20) | 0.635 |
| Fibrinogen, g/L | 2.62 (2.21, 3.08) | 2.71 (2.28, 3.14) | 2.48 (2.11, 2.95) | <0.001 |
| D-dimer | 3015 (869, 7220) | 1799 (544, 3996) | 5688 (2481, 11879) | <0.001 |
| FDP | 23.45 (7.43, 64.67) | 13.00 (4.71, 36.36) | 50.01 (18.34, 107.97) | <0.001 |
| WBC | 12.32 (9.48, 15.81) | 11.27 (8.68, 14.22) | 14.09 (10.81, 17.59) | <0.001 |
| Neutrophils, % (valid n=639) | 87.50 (81.80, 90.90) | 85.90 (78.40, 90.25) | 88.70 (84.80, 91.50) | <0.001 |
| Hemoglobin, g/L | 132.00 (119.00, 144.00) | 133.00 (120.50, 147.00) | 130.00 (118.00, 141.00) | 0.022 |
| Platelet count | 195.00 (155.75, 236.00) | 205.00 (163.00, 245.00) | 188.00 (148.00, 224.00) | <0.001 |
| **CT timing and injury characteristics** | | | | |
| Time to baseline CT, h | 2.00 (1.50, 2.00) | 2.00 (1.50, 2.00) | 1.50 (1.50, 2.00) | <0.001 |
| Time to follow-up CT, h | 23.00 (17.00, 42.50) | 24.50 (18.00, 46.50) | 22.00 (16.00, 37.00) | <0.001 |
| Baseline-to-follow-up CT interval, h | 21.50 (15.50, 41.00) | 22.50 (16.00, 44.50) | 20.50 (14.50, 35.50) | 0.001 |
| Baseline hematoma volume, mL | 3.18 (1.70, 6.16) | 2.98 (1.70, 5.51) | 3.45 (1.65, 7.11) | 0.207 |
| Cerebral contusion, n (%) |  |  |  | <0.001 |
| No | 227 (35.47) | 205 (57.75) | 22 (7.72) |  |
| Yes | 413 (64.53) | 150 (42.25) | 263 (92.28) |  |
| Epidural hematoma, n (%) |  |  |  | 0.287 |
| No | 479 (74.84) | 272 (76.62) | 207 (72.63) |  |
| Yes | 161 (25.16) | 83 (23.38) | 78 (27.37) |  |
| Diffuse axonal injury, n (%) |  |  |  | 0.004 |
| No | 589 (92.03) | 337 (94.93) | 252 (88.42) |  |
| Yes | 51 (7.97) | 18 (5.07) | 33 (11.58) |  |
| Subdural hematoma, n (%) |  |  |  | <0.001 |
| No | 270 (42.19) | 192 (54.08) | 78 (27.37) |  |
| Yes | 370 (57.81) | 163 (45.92) | 207 (72.63) |  |
| Subarachnoid hemorrhage, n (%) |  |  |  | <0.001 |
| No | 190 (29.69) | 143 (40.28) | 47 (16.49) |  |
| Yes | 450 (70.31) | 212 (59.72) | 238 (83.51) |  |
| Long-bone fracture, n (%) |  |  |  | 0.004 |
| No | 470 (73.44) | 277 (78.03) | 193 (67.72) |  |
| Yes | 170 (26.56) | 78 (21.97) | 92 (32.28) |  |
| Skull fracture, n (%) |  |  |  | <0.001 |
| No | 228 (35.62) | 163 (45.92) | 65 (22.81) |  |
| Yes | 412 (64.38) | 192 (54.08) | 220 (77.19) |  |
| Frontal lobe involvement, n (%) |  |  |  | <0.001 |
| Diffuse/bilateral | 156 (24.38) | 47 (13.24) | 109 (38.25) |  |
| Left | 121 (18.91) | 54 (15.21) | 67 (23.51) |  |
| None | 208 (32.50) | 159 (44.79) | 49 (17.19) |  |
| Right | 155 (24.22) | 95 (26.76) | 60 (21.05) |  |
| Parietal lobe involvement, n (%) |  |  |  | <0.001 |
| Diffuse/bilateral | 50 (7.81) | 20 (5.63) | 30 (10.53) |  |
| Left | 116 (18.12) | 39 (10.99) | 77 (27.02) |  |
| None | 366 (57.19) | 236 (66.48) | 130 (45.61) |  |
| Right | 108 (16.88) | 60 (16.90) | 48 (16.84) |  |
| Occipital lobe involvement, n (%) |  |  |  | 0.013 |
| Diffuse/bilateral | 10 (1.56) | 5 (1.41) | 5 (1.75) |  |
| Left | 77 (12.03) | 32 (9.01) | 45 (15.79) |  |
| None | 492 (76.88) | 290 (81.69) | 202 (70.88) |  |
| Right | 61 (9.53) | 28 (7.89) | 33 (11.58) |  |
| Temporal lobe involvement, n (%) |  |  |  | <0.001 |
| Diffuse/bilateral | 128 (20.00) | 40 (11.27) | 88 (30.88) |  |
| Left | 150 (23.44) | 75 (21.13) | 75 (26.32) |  |
| None | 173 (27.03) | 125 (35.21) | 48 (16.84) |  |
| Right | 189 (29.53) | 115 (32.39) | 74 (25.96) |  |
| Deep structure involvement, n (%) |  |  |  | 0.022 |
| No | 510 (79.69) | 295 (83.10) | 215 (75.44) |  |
| Yes | 130 (20.31) | 60 (16.90) | 70 (24.56) |  |

Values are median (IQR) for continuous variables and n (%) for categorical variables. P values were calculated using the Mann–Whitney U test for continuous variables and Pearson’s chi-square test or Fisher’s exact test for categorical variables, as appropriate.

For timing variables, values >48 h were truncated at 48 h. Four non-positive CT intervals were replaced with the HE-stratum mean after truncation (23.59 h). Abbreviations: APTT, activated partial thromboplastin time; CT, computed tomography; DAI, diffuse axonal injury; FDP, fibrin degradation products; GCS, Glasgow Coma Scale; HE, hematoma expansion; PT, prothrombin time; WBC, white blood cell count.

**Supplementary Table 2. Multivariable logistic regression results of the baseline model and the nested HE model for predicting favorable 6-month functional outcome in the overall cohort**

**Panel A. Baseline model**

| **Variable** | **β** | **SE** | **Z** | **P value** | **OR (95% CI)** |
| --- | --- | --- | --- | --- | --- |
| Intercept | 1.69 | 0.85 | 1.99 | 0.047 | 5.42 (1.02–28.71) |
| Cerebral contusion, present | −0.76 | 0.35 | −2.15 | 0.031 | 0.47 (0.23–0.93) |
| Diffuse axonal injury, present | −1.03 | 0.44 | −2.33 | 0.020 | 0.36 (0.15–0.85) |
| Long-bone fracture, present | −1.18 | 0.30 | −3.91 | <0.001 | 0.31 (0.17–0.56) |
| Frontal involvement, left | 0.49 | 0.38 | 1.29 | 0.196 | 1.63 (0.78–3.41) |
| Frontal involvement, none | 1.26 | 0.42 | 2.97 | 0.003 | 3.51 (1.53–8.05) |
| Frontal involvement, right | 0.26 | 0.36 | 0.70 | 0.481 | 1.29 (0.63–2.64) |
| Age, per year | −0.05 | 0.01 | −4.40 | <0.001 | 0.95 (0.93–0.97) |
| GCS score, per point | 0.36 | 0.04 | 8.55 | <0.001 | 1.43 (1.32–1.55) |
| D-dimer, per unit | −0.01 | 0.00 | −3.61 | <0.001 | 0.99 (0.99–0.99) |
| Baseline hematoma volume, per mL | −0.15 | 0.03 | −4.53 | <0.001 | 0.86 (0.80–0.92) |

**Panel B. Nested HE model**

| **Variable** | **β** | **SE** | **Z** | **P value** | **OR (95% CI)** |
| --- | --- | --- | --- | --- | --- |
| Intercept | 2.14 | 0.88 | 2.42 | 0.015 | 8.49 (1.50–47.96) |
| Cerebral contusion, present | 0.13 | 0.41 | 0.32 | 0.750 | 1.14 (0.51–2.56) |
| Diffuse axonal injury, present | −0.55 | 0.46 | −1.21 | 0.225 | 0.58 (0.24–1.41) |
| Long-bone fracture, present | −1.11 | 0.30 | −3.68 | <0.001 | 0.33 (0.18–0.59) |
| Frontal involvement, left | 0.37 | 0.38 | 0.97 | 0.335 | 1.44 (0.68–3.05) |
| Frontal involvement, none | 0.95 | 0.44 | 2.17 | 0.030 | 2.60 (1.10–6.15) |
| Frontal involvement, right | −0.02 | 0.38 | −0.05 | 0.964 | 0.98 (0.47–2.05) |
| Age, per year | −0.05 | 0.01 | −4.20 | <0.001 | 0.96 (0.94–0.98) |
| GCS score, per point | 0.34 | 0.04 | 8.10 | <0.001 | 1.41 (1.30–1.53) |
| D-dimer, per unit | −0.01 | 0.00 | −2.85 | 0.004 | 0.99 (0.99–0.99) |
| Baseline hematoma volume, per mL | −0.17 | 0.03 | −5.07 | <0.001 | 0.84 (0.79–0.90) |
| Hematoma expansion, present | −1.60 | 0.38 | −4.25 | <0.001 | 0.20 (0.10–0.42) |

Multivariable logistic regression models predicted favorable 6-month functional outcome. The nested HE model was constructed by adding hematoma expansion to the baseline model. For all binary Yes/No variables, “No” was used as the reference category. Diffuse/bilateral frontal involvement was used as the reference category for frontal involvement. Odds ratios (ORs) with 95% confidence intervals (CIs) are presented. An OR >1 indicates a higher likelihood of favorable outcome, whereas an OR <1 indicates a lower likelihood of favorable outcome.

**Abbreviations:** CI, confidence interval; GCS, Glasgow Coma Scale; HE, hematoma expansion; OR, odds ratio; SE, standard error.

**Supplementary Table 3 Detailed comparison of the baseline model and the nested HE model in the overall cohort**

| Comparison | AIC (baseline model) | AIC (nested HE model) | ΔAIC | AUC (baseline model) | AUC (nested HE model) | ΔAUC | LRT χ² (df = 1) | LRT P value | DeLong Z | DeLong P value |
| --- | --- | --- | --- | --- | --- | --- | --- | --- | --- | --- |
| Baseline model vs nested HE model | 392.767 | 365.908 | 26.859 | 0.920 | 0.929 | 0.009 | 28.859 | <0.001 | 2.184 | 0.0289 |

Detailed model comparison between the baseline model and the nested HE model in the overall cohort. Comparison metrics included the Akaike information criterion (AIC), change in AIC (ΔAIC), likelihood ratio test (LRT), area under the receiver operating characteristic curve (AUC), change in AUC (ΔAUC), and the DeLong test. The nested HE model was obtained by adding hematoma expansion to the baseline model. **Abbreviations:** AIC, Akaike information criterion; LRT, likelihood ratio test; AUC, area under the receiver operating characteristic curve; HE, hematoma expansion.

**Supplementary Table 4 Best-subset sensitivity analysis for baseline model specification in the overall cohort**

| **Model** | **Selection criterion** | **Variables retained** | **AIC** | **BIC** | **AUC** | **ΔAUC after adding HE** |
| --- | --- | --- | --- | --- | --- | --- |
| Baseline model | Primary analysis | Age; GCS score; D-dimer; baseline hematoma volume; cerebral contusion; diffuse axonal injury; long-bone fracture; frontal lobe involvement | 388.498 | 437.574 | 0.920 | 0.009 |
| AIC-selected best-subset model | AIC | Age; GCS score; D-dimer; baseline hematoma volume; cerebral contusion; diffuse axonal injury; long-bone fracture; frontal lobe involvement | 388.498 | 437.574 | 0.920 | 0.009 |
| BIC-selected best-subset model | BIC | Age; GCS score; D-dimer; baseline hematoma volume; long-bone fracture | 399.867 | 426.636 | 0.909 | 0.017 |

Best-subset selection was performed in the overall cohort of 640 patients. Candidate baseline models were compared using AIC, BIC, and AUC. HE was then added to each selected model, and ΔAUC indicates the increase in AUC after adding HE. **Abbreviations:** AIC, Akaike information criterion; AUC, area under the receiver operating characteristic curve; BIC, Bayesian information criterion; GCS, Glasgow Coma Scale; HE, hematoma expansion.

**Supplementary Table 5 Timing-adjusted sensitivity analysis for favorable 6-month functional outcome**

**Panel A. Multivariable logistic regression with forced adjustment for baseline-to-follow-up CT interval (n = 640)**

| **Variable** | **β** | **SE** | **OR (95% CI)** | **P value** |
| --- | --- | --- | --- | --- |
| Age, per year | -0.045 | 0.011 | 0.96 (0.94–0.98) | <0.001 |
| GCS score, per point | 0.326 | 0.043 | 1.39 (1.27–1.51) | <0.001 |
| D-dimer, per unit | -0.00004 | 0.00001 | 0.99996 (0.99994–0.99999) | 0.002 |
| Baseline hematoma volume, per mL | -0.180 | 0.034 | 0.84 (0.78–0.89) | <0.001 |
| Cerebral contusion: yes vs no | 0.133 | 0.421 | 1.14 (0.50–2.61) | 0.752 |
| Diffuse axonal injury: yes vs no | -0.467 | 0.455 | 0.63 (0.26–1.53) | 0.304 |
| Frontal involvement: left vs diffuse/bilateral | 0.380 | 0.385 | 1.46 (0.69–3.11) | 0.324 |
| Frontal involvement: none vs diffuse/bilateral | 0.919 | 0.438 | 2.51 (1.06–5.92) | 0.036 |
| Frontal involvement: right vs diffuse/bilateral | 0.028 | 0.377 | 1.03 (0.49–2.15) | 0.940 |
| Long-bone fracture: yes vs no | -1.136 | 0.307 | 0.32 (0.18–0.59) | <0.001 |
| Baseline-to-follow-up CT interval, per hour | -0.028 | 0.010 | 0.97 (0.95–0.99) | 0.007 |
| Hematoma expansion: yes vs no | -1.841 | 0.400 | 0.16 (0.07–0.35) | <0.001 |

**Panel B. Comparison of the timing-adjusted baseline and timing-adjusted HE models**

| **Metric** | **Timing-adjusted baseline model** | **Timing-adjusted HE model** | **Difference / test** | **P value** |
| --- | --- | --- | --- | --- |
| AIC | 387.439 | 365.236 | ΔAIC = 22.203 |  |
| AUC (95% CI) | 0.920 (0.895–0.946) | 0.933 (0.910–0.956) | ΔAUC = 0.012 | 0.006 |
| Likelihood-ratio test | - | - | χ² = 24.203 (df = 1) | <0.001 |
| DeLong test | - | - | Z = 2.766 | 0.006 |

The models predicted favorable 6-month functional outcome (GOS 4–5). The CT interval was forced into both models as a design/observation-time covariate and was not subjected to variable selection. Values >48 h were truncated at 48 h, and four non-positive CT intervals were replaced with the HE-stratum mean. Diffuse/bilateral frontal involvement and absence of HE, cerebral contusion, diffuse axonal injury, and long-bone fracture were reference categories.

**Abbreviations:** AIC, Akaike information criterion; AUC, area under the receiver operating characteristic curve; CI, confidence interval; CT, computed tomography; GCS, Glasgow Coma Scale; HE, hematoma expansion; OR, odds ratio; SE, standard error.

**Supplementary Table 6 Predictive performance of the HE-positive subgroup model for favorable 6-month functional outcome**

| **AUC (95% CI)** | **Accuracy (95% CI)** | **Sensitivity (95% CI)** | **Specificity (95% CI)** | **PPV (95% CI)** | **NPV (95% CI)** | **Optimal cutoff** |
| --- | --- | --- | --- | --- | --- | --- |
| 0.89 (0.85-0.93) | 0.83 (0.78-0.87) | 0.85 (0.79 - 0.90) | 0.80 (0.73 - 0.87) | 0.86 (0.81 - 0.91) | 0.78 (0.71 - 0.86) | 0.544 |

Predictive performance of the multivariable model developed in HE-positive patients for favorable 6-month functional outcome. Performance metrics are presented as point estimates with 95% confidence intervals, and were calculated at the optimal cutoff determined by the Youden index. **Abbreviations**: AUC, area under the receiver operating characteristic curve; PPV, positive predictive value; NPV, negative predictive value; CI, confidence interval; HE, hematoma expansion.

**Supplementary Fig. 1 Representative images of manual hematoma segmentation on serial head CT scans**

Representative examples of region-of-interest-based manual hematoma segmentation on the first two in-hospital head CT scans used for HE assessment. The follow-up CT was completed within 48 h after injury onset. Colored overlays indicate the segmented hemorrhagic lesions used for hematoma volume measurement in 3D Slicer. (A) Baseline head CT of a representative patient with traumatic intracranial hemorrhage. (B) Follow-up head CT of the same patient showing increased hemorrhage volume, meeting the definition of hematoma expansion. (C) Baseline head CT of another representative patient with traumatic intracranial hemorrhage. (D) Follow-up head CT of the same patient showing a newly detected hemorrhagic lesion, meeting the definition of hematoma expansion.

**Supplementary Fig. 2 Decision curve analysis of the nested HE model for predicting favorable 6-month functional outcome in the overall cohort**

Decision curve analysis of the nested HE model in the overall cohort. The x-axis indicates threshold probability, and the y-axis indicates net benefit. The clinical utility of the nested HE model was compared with the default strategies of treating all patients and treating no patients.

**Supplementary Fig. 3 Bootstrap internal validation calibration curve of the nested HE model for predicting favorable 6-month functional outcome in the overall cohort**

Bootstrap internal validation calibration plot of the nested HE model based on 1000 resamples in the overall cohort. Predicted probability is shown on the x-axis and observed probability on the y-axis. The figure presents the optimism-corrected calibration of the model after internal validation.
